# Vesiculobullous eruption of the right arm after intravenous clarithromycin

**DOI:** 10.4103/0253-7613.75679

**Published:** 2011-02

**Authors:** Abdulkadir Kuçukbayrak, Engin Senel, Zeynep Seckin Kücükbayrak, Ersin Gunay, Ezgi Simsek

**Affiliations:** Clinic of Infectious Diseases and Clinical Microbiology, Atatürk Chest Diseases and Thoracic Surgery Training and Research Hospital, Ankara, Turkey; 1 Clinic of Dermatology, Çankiri State Hospital, Çankiri, Turkey; 2 Department of Physiology, Duzce University School of Medicine, Duzce, Turkey; 3 Clinic of Chest Diseases, Atatürk Chest Diseases and Thoracic Surgery Training and Research Hospital, Ankara, Turkey

**Keywords:** Adverse drug reaction, bullous reaction, clarithromycin, intravenous, venous thrombosis

## Abstract

Clarithromycin is a macrolide antibiotic. In clinical trials, adverse drug reactions of clarithromycin are usually mild and transient. Only 1% of the adverse reactions are severe. Herein, we present a case with vesiculobullous skin reaction and vein thrombosis caused by administration of intravenous clarithromycin.

## Introduction

Clarithromycin is a macrolide antibiotic. It is highly active against gram-positive bacteria and has a good activity against Haemophilus influenzae, Moraxella catarrhalis and atypical bacteria.[1]

In clinical studies, adverse drug reactions (ADRs) of clarithromycin are usually mild and transient. Only 1% of the adverse reactions are severe.[1] The most common ADRs observed after intravenous clarithromycin treatment are phlebitis, pain and inflammation, which are mild and reversible.[2] We present a case of vesiculobullous eruption and vein thrombosis caused by the intravenous administration of clarithromycin.

## Case Report

A 73-year-old man presented to the emergency room with complaints of cough and purulent sputum. He had a history of cerebrovascular disease 1 year ago. His family history was unremarkable.

Physical examination revealed decreased lung sounds in the left hemithorax. Complete blood count, liver and kidney functional laboratory tests were in the normal range. The patient was hospitalized. Pulse intravenous clarithromycin (500 mg twice-daily) treatment was initiated due to lower respiratory tract infection. No concomitant or additional medication was given. In 12 h of the treatment, erythema and swelling developed on his right forearm where i.v. clarithromycin was given.

**Figure 1:**
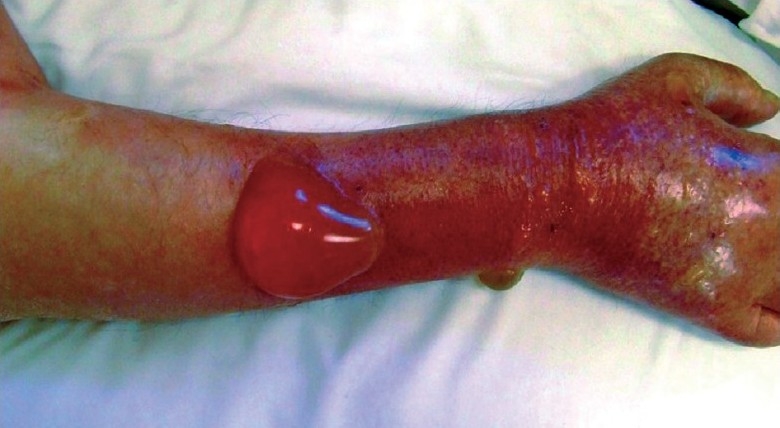
Edema, erythema and bullae on the right arm after pulse intravenous clarithromycin application

**Figure 2:**
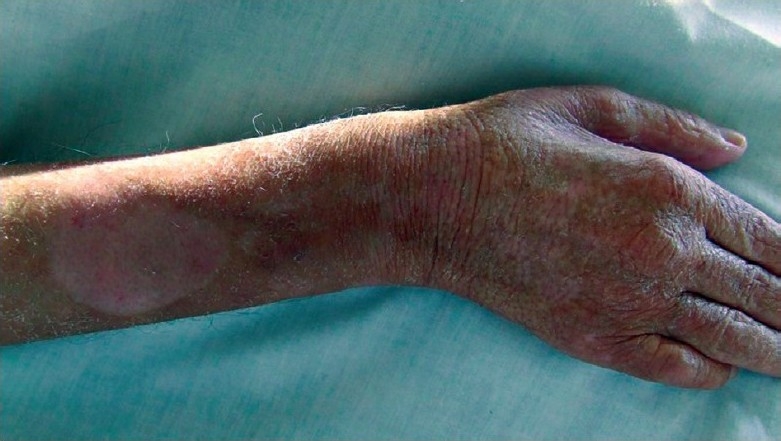
Residual scar 1 month after the presentation of the patient

Dermatological examination revealed sharp erythema that spread from the middle of the forearm to the entire right dorsal hand, with multiple vesicles and bullae filled with serous material [Figure 1]. Arterial Doppler ultrasonography of the right arm was normal. Venous Doppler ultrasonography revealed venous thrombosis and edema in subcutaneous tissue on the right arm. Intravenous clarithromycin was stopped. Intravenous piperacillin-tazobactam treatment (3 × 4.5 g daily) was initiated for the lower tract infection and conservative local wound care treatment with analgesics and dressings was administrated. At the end of the first month of follow-up, the clinical findings improved [Figure 2].

## Discussion

Clarithromycin-associated adverse reactions are wide ranging, from mild urticaria and skin eruptions to rare conditions such as anaphylaxis, Stevens-Johnson syndrome and toxic epidermal necrolysis.[3] Vorbach *et al*. suggested that clarithromycin has a better endothelial compatibility when it dilutes to a final concentration of 1 mg/ml. It was previously reported that the development of clarithromycin-induced phlebitis could be reduced by manufacturers with production of the diluted form (1 mg/ml) of clarithromycin.[4]

The Naranjo criteria is frequently used for determination of causality for suspected ADRs.[5] A causality assessment of this ADR using the Naranjo criteria revealed that an adverse drug event due to clarithromycin was possible in this case (overall score, 4).

To conclude, intravenous pulse administration of clarithromycin should not be performed. Local ADRs depend on the infusion time and the drug concentration. We recommend that slow infusion and low concentrations can reduce the frequency and severity of the local ADRs. Although local adverse reactions may be serious, they can be improved with close observation and appropriate treatment.
